# Long-term management of recurrent papillary thyroid carcinoma treated with lenvatinib for over 5 years: a case report

**DOI:** 10.1186/s40792-022-01374-0

**Published:** 2022-01-27

**Authors:** Tsuneo Imai, Hironobu Kobayashi, Tetsu Senaha, Toshiaki Imaizumi, Yoshiharu Murata

**Affiliations:** 1 Kachi Memorial Hospital, 456 Fujimi, Minami-ohshimizu-cho, Toyohashi, Aichi 441-8555 Japan; 2 Department of Breast and Endocrine Surgery, Higashinagoya National Hospital, National Hospital Organization, 5-101 Umemorizaka, Meito-ku, Nagoya, Aichi 465-8620 Japan; 3 Department of Breast and Endocrine Surgery, Nagoya Central Hospital, 3-3-3 Taiko, Nakamura-ku, Nagoya, Aichi 453-0801 Japan

**Keywords:** Adrenal metastasis, Bone metastasis, Lenvatinib, Long-term PR, Lung metastasis, Lymph node metastasis, Papillary thyroid cancer, RAI refractory, Recurrence, Reversible posterior leukoencephalopathy syndrome, Tyrosine kinase inhibitor

## Abstract

**Background:**

Few reports exist of the long-term management of recurrent and progressive papillary thyroid carcinoma (PTC) with a tyrosine kinase inhibitor for over 5 years.

**Case presentation:**

A 57-year-old woman was referred to a psychiatric hospital for the treatment of schizophrenia. The patient had been diagnosed with a PTC at the age of 40 and subsequently underwent a left thyroid lobectomy. At 47, completion total thyroidectomy and lymph node dissection were performed and the patient assessed as radioactive iodine refractory postoperatively. External radiation therapy was performed for Rouviere lymph nodes. At 57, neck and mediastinal lymph nodes, and lung metastases had progressed, and the trachea became narrowed by para-tracheal lymph node compression. After 2 weeks of sorafenib therapy on an outpatient basis, the patient was discovered unconsciousness at home and transferred to hospital by ambulance; sorafenib therapy was stopped. The patient was diagnosed with reversible posterior leukoencephalopathy syndrome by brain magnetic resonance imaging. External radiation therapy to the site of the tracheal stenosis in the neck and mediastinum was performed. The patient’s mental symptoms worsened, and she was referred to a psychiatric hospital, Kachi Memorial Hospital, in July 2015. In September, the patient’s mental state stabilized and in November, after computed tomography revealed rapid disease progression, lenvatinib was commenced at a daily dose of 24 mg. Measurable solid recurrence sites were neck lymph nodes in the pre-laryngeal subcutaneous space, right lobe of the lung, and left adrenal. After 3 months, the tumors shrank in a partial response (PR). Because of several adverse events, occasional dose reductions or discontinuations of lenvatinib were sometimes necessary. Since re-starting lenvatinib, treatment with this for 51 consecutive months was achieved while maintaining a PR. Although a new bone metastasis was noted after 57 months of lenvatinib, treatment was continued for another 9 months. The patient subsequently passed away in June 2021.

**Conclusions:**

The long-term treatment of recurrent PTC with lenvatinib was feasible, with manageable adverse events, for more than 5 years.

## Background

After the initial clinical application of tyrosine kinase inhibitors (TKIs), these became a standard systemic treatment for radioactive iodine (RAI) refractory, progressive, and metastatic or locally advanced differentiated thyroid cancer. Two TKIs have been widely used clinically: sorafenib, which was approved by the US Food and Drug Administration in 2014 after it was found to be effective in a phase 3 DECISION study [1]; and lenvatinib in 2015 according to the results of a phase 3 SELECT study [2]. Both TKIs have various adverse events, some of which are serious. Therefore, in Japan, the indication and timing of the use of TKIs are strictly regulated according to various settings, such as being based on a specific institutional decision, academic society, or medical instruction under Japanese health insurance. Patient experiences and outcomes from the use of TKIs have been reported from high-volume centers such as a Cancer Center or University Hospital [3, 4]. In these reports, the duration of most TKI treatments was from 1 to 3 years [5, 6]. In the case of general hospitals, physicians are not sufficiently specialized to manage TKIs or treat thyroid cancer. Therefore, even if TKIs are indicated for patients with recurrent and progressive papillary thyroid cancer (PTC), it is likely that the administration of TKIs is discontinued early. In this report, we present a case of metastatic, recurrent, progressive PTC in which lenvatinib was shown to be markedly effective for more than 5 years after several treatments, such as surgery, external radiations, and sorafenib.

## Case presentation

A 57-year-old woman (height, 164 cm; weight, 50 kg) was referred to Kachi Memorial Hospital, a psychiatric hospital, for the treatment of schizophrenia. An initial diagnosis of schizophrenia was made at the age of 18, and the patient was later repeatedly treated in several psychiatry hospitals. The patient was found to have PTC at the age of 40 and a left thyroid lobectomy was subsequently performed at Aichi Cancer Center Hospital. At 47 years of age, the patient showed pulmonary metastases and lymph node recurrences, and a completion total thyroidectomy and lymph node dissection were performed. After the operation, a whole-body scan with 10 mCi of RAI revealed no uptake in both pulmonary metastases and residual parapharyngeal metastases. An expert panel concluded there was no indication for high-dose RAI therapy and thus external radiation therapy was performed for Rouviere parapharyngeal lymph nodes. At 57 years of age, the patient showed metastases to cervical and mediastinal lymph nodes as well as lung metastases that had progressed. The trachea became narrowed by a para-tracheal metastatic lymph node compression. Therefore, sorafenib was started on an outpatient basis. After 2 weeks of sorafenib therapy, the patient was discovered unconscious at home and transferred to hospital by ambulance. Sorafenib therapy was then stopped. The patient was subsequently diagnosed with reversible posterior leukoencephalopathy syndrome (RPLS) by brain magnetic resonance imaging (MRI). External radiation therapy to the site of a tracheal stenosis in the neck and mediastinum was performed. However, the patient’s mental symptoms worsened, and she was referred and transferred to Kachi Memorial Hospital in July 2015.

**Fig. 1:**
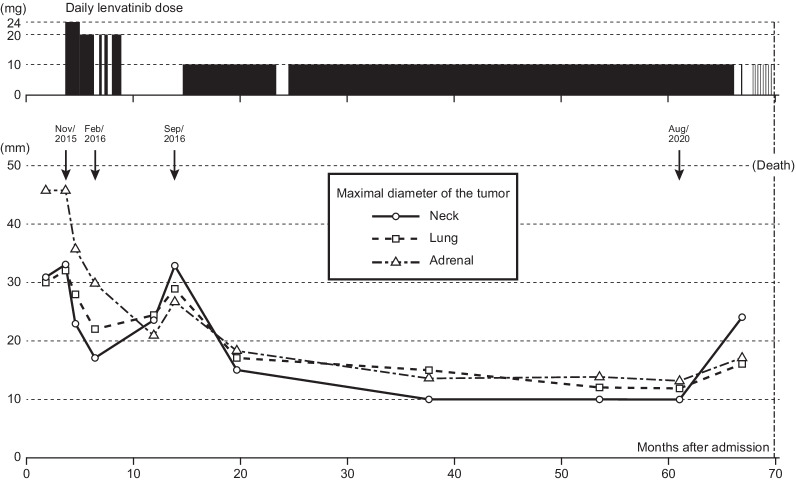
A series of measurable tumor sizes and lenvatinib doses over time. Lenvatinib dose and duration of treatment are shown in the upper part of the graph (black boxes). The size of the three measurable sites (neck: pre-laryngeal lymph node, lung: largest mass in the right lobe, adrenal: left) are represented by dashed or solid lines with open circles, boxes, and triangles, respectively. Four arrows indicate the dates of computed tomography (CT) scans shown in Fig. 2

Two months after hospital admission, the patient’s mental state stabilized. Although Kachi Memorial Hospital is a psychiatric hospital, the director is an endocrinologist and specialist in thyroid diseases. Furthermore, two endocrine surgeons worked at the hospital on a part-time basis. Therefore, the stage of thyroid cancer was evaluated and a treatment strategy formulated. According to computed tomography (CT), the largest recurrence sites were in cystic lymph nodes in the right para-tracheal space beyond the neck to the mediastinum. In addition, measurable solid recurrence sites existed, such as neck lymph nodes in the pre-laryngeal subcutaneous space, the right lobe of lung, and the left adrenal. A follow-up evaluation by CT after 2 months revealed such tumors to be enlarged (Fig. 1). After 2 months’ treatment for schizophrenia, the patient’s mental condition was good: she was calm and intelligent enough to spend most of the day reading English books written by Shakespeare, for example. However, the PTC was considered progressive enough to become lethal in the near future if an effective therapy was not tried. Therefore, we concluded that lenvatinib treatment should be started immediately. We explained the lenvatinib treatment to the patient including any possible adverse events. Accordingly, the patient agreed and gave written, informed consent.

Lenvatinib was commenced at a daily dose of 24 mg in November 2015. Other medications were 20 mg olanzapine, 4 mg risperidone, 3 mg flunitrazepam, 1 mg clonazepam, 100 μg levothyroxine sodium hydrate, 0.5 μg alfacalcidol, 48 μg lubiprostone, 1500 mg magnesium oxide, and 36 mg sennoside, per day, respectively. Hypertension was managed with daily 5 mg amlodipine besilate, and 20 mg azilsartan. Hand–foot syndrome with fever developed twice, and required a repeated discontinuation or a dose reduction of lenvatinib on each occasion (Fig. 1).

**Fig. 2:**
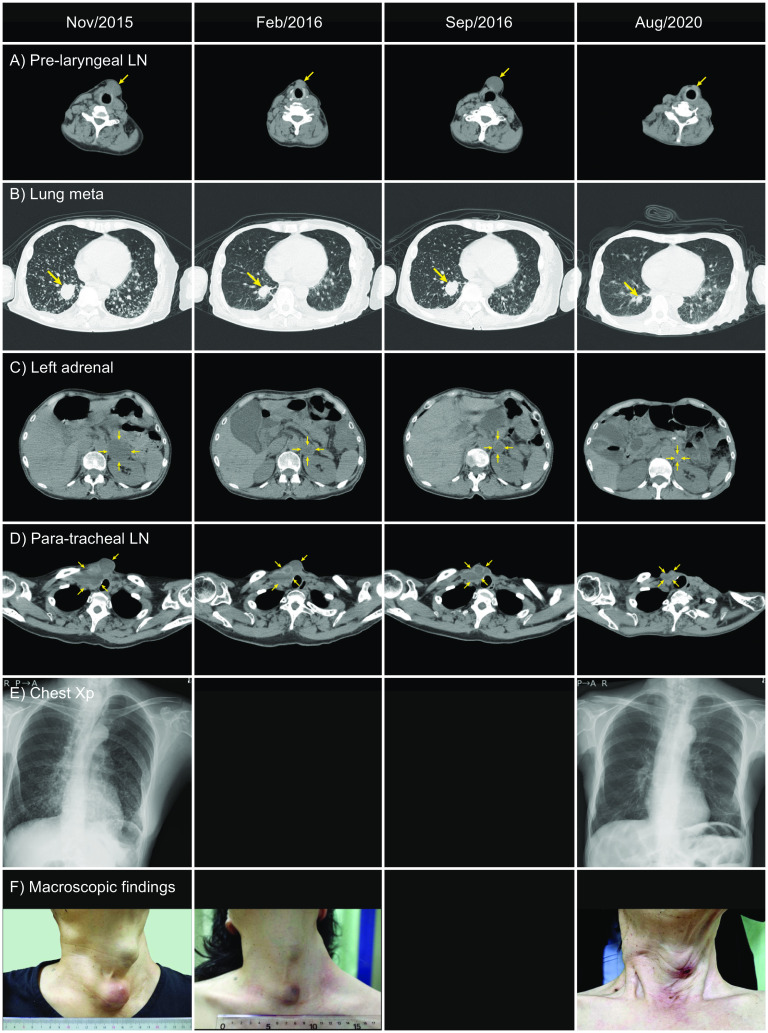
Changes in images from computed tomography (CT) scans (**A**–**D**) and chest X-rays (Xp) (**E**), and macroscopic appearances of the patient’s neck (**F**) during her clinical course. Enlarged pre-laryngeal lymph node (LN), lung metastasis, left adrenal metastasis, and cystic lymph nodes in the neck and mediastinum are shown in **A**–**D**, respectively

Before starting on lenvatinib, measurable solid recurrence sites for the cancer were noted in the neck lymph nodes in the pre-laryngeal subcutaneous space, right lobe of lung, and left adrenal, with maximal diameters of 33 mm, 32 mm, and 46 mm, respectively (Fig. 2, Nov/2015, A–C). Within 3 months after lenvatinib administration, the sum of tumor sizes was reduced by more than 30% and, thus, this was assessed as a partial response (PR) according to Response Evaluation Criteria in Solid Tumors (RECIST) version 1.1 ([7], Fig. 2, Feb/2016, A–C). After 5 months, the neck cystic lesion ruptured spontaneously, and a small amount of bleeding occurred intermittently. Therefore, lenvatinib was stopped for 5 months.

During the discontinuation of lenvatinib, the tumors became enlarged, and neck lymph nodes and lung metastases regrew to almost 100% of the pre-treatment size (Fig. 1, Fig. 2, Sep/2016, A–C). Pre-laryngeal lymph node, lung, and adrenal tumors were not attached to large vessels, and the para-tracheal lymph nodes were mainly cystic. Thus, the endocrine team assessed that the risk of massive bleeding would be low, and lenvatinib was restarted at a dose of 10 mg a day in October 2016. After resuming lenvatinib, the patient showed only a few adverse events and therefore the medication was continued for another four and half years. However, it was discontinued in 2017 when her psychological condition worsened. After an improvement in psychological state, lenvatinib was restarted again after one month. Measurable tumors continued to be reduced in size, and a PR was observed until August 2020 (Figs. 1, 2 Aug/2020, A–C). However, in August 2020, a new bone metastasis in the sternum was found by CT scan (data not shown). Fortunately, the symptoms of bone metastasis were absent, and lenvatinib was continued despite progressive disease. Other than measurable lesions, cystic lymph nodes in the neck and mediastinum, and millions of tiny lung metastases, were also markedly reduced during lenvatinib treatment (Fig. 2D–F).

In February 2021, tumors in the neck and mediastinum, including the sternum, became enlarged, and the patient occasionally developed aspiration pneumonia. Lenvatinib was discontinued and restarted several times, being administered twice a week (10 mg/day) from April 2021 onwards. However, the multiple tumors progressively grew, and the patient’s general condition became worse, with multiple, large, cystic bone metastases evident. The patient subsequently passed away in June 2021.

## Discussion

Since publication of the SELECT trial, which was subsequently approved in USA, Europe, and Japan for the treatment of RAI refractory and progressive differentiated thyroid carcinoma in 2015, lenvatinib has been widely used in clinical practice [2]. The SELECT trial demonstrated an improvement in progression-free survival (PFS) with lenvatinib compared to placebo of 18.3 vs. 3.6 months. Interestingly, in a prespecified sub-analysis of the SELECT trial, improved overall survival (OS) was found in lenvatinib-treated patients compared with OS in placebo patients over 65 years of age [8]. An updated analysis of the SELECT trial showed that patients with a complete or partial response to lenvatinib achieved a median PFS of 33.1 months [9].

In clinical practice, lenvatinib tends to be used in patients with more advanced tumor stages, who have been more heavily pretreated, are sicker, or have more comorbidities, and who are not suitable candidates for trials. Indeed, several reports exist of lenvatinib treatment for advanced thyroid carcinoma [4, 10–16]. In these case reports, the median lenvatinib treatment was 6–27 months and the longest was 64 months. In the current report, the patient was treated for 66 months from start to finish, though a tentative discontinuation of lenvatinib was required at the beginning of her therapy.

A requirement exists within a clinical trial for treatment to be stopped when progressive disease is noted. However, in real-world practice, when no other effective treatment exists, therapy is allowed to continue if drug toxicity is manageable. In the present case, lenvatinib treatment was maintained after a new bone metastasis was noted since adverse events were minimal and lesions such as those in the lung, lymph nodes, and adrenal metastases could be controlled by lenvatinib. Accordingly, lenvatinib treatment beyond progressive disease lasted for a further 9 months until just before the patient’s death.

Lenvatinib was the sole remaining therapy available to this patient after multiple treatments, such as surgeries, external radiations, and sorafenib, were tried. Since sorafenib was discontinued because of severe adverse events (i.e., RPLS), we did not re-challenge with sorafenib in the present case.

PTC is normally defined as RAI refractory when high-dose RAI treatment has no effect on metastatic lesions. However, the patient was never treated with high-dose RAI therapy since her cancer was assessed as being RAI refractory from the results of RAI scintigraphy after administering small amounts of RAI. This may not be a standard treatment, globally, but is a common practice in many cities in Japan. High-dose RAI therapy is not performed for many patients with recurrent differentiated thyroid cancer in Japan due to the very limited number of facilities available for high-dose RAI therapy since the use of radiation is unpopular for historical reasons [17, 18]. Furthermore, there was a report that the diagnostic RAI scintigraphy using small amounts of RAI can predict the efficacy of high-dose RAI therapy [19]. This was the reason why high-dose RAI therapy was not performed in the present case. In addition, Nakanishi et al. reported a low prevalence of RAI uptake by metastatic lesions of PTC in patients older than 55 years [20]. From this point of view, it was applicable in the present case that the patient was given lenvatinib omitting high-dose RAI therapy.

In 2015 when we saw the patient at Kachi Memorial Hospital, we evaluated her thyroid cancer as becoming progressively worse and predicted that she would die within one year. Indeed, the median OS of a patient with lung metastases of > 2.0 cm was reported as 19.3 months in the placebo group in a phase 3 trial (SELECT) (21). Of note, in this SELECT trial, crossover from placebo to lenvatinib was allowed. As a matter of fact, 89% of patients of the placebo arm took lenvatinib after a judgment of progressive disease. If any of the patients had not taken lenvatinib, the OS would have been much shorter than 19.3 months. Because the patient in this report had multiple lung metastases of > 3.0 cm, her prognosis was very poor without lenvatinib. Considering that the patient lived for 66 months from the initiation of lenvatinib therapy without any severe adverse events, such treatment not only allowed her to survive longer, but also improved her quality of life. Therefore, the outcome of treatment with lenvatinib in the present case should shed light upon the prognosis of patients with differentiated thyroid cancer, and multiple and sizable lung metastases.

## Conclusions

Long-term lenvatinib treatment of recurrent PTC was feasible, with manageable adverse events, for more than 5 years.

## Data Availability

Not applicable.

## Abbreviations

CT: Computed tomography
OS: Overall survival
PFS: Progression-free survival
PR: Partial response
PTC: Papillary thyroid cancer
RAI: Radioactive iodine
RECIST: Response Evaluation Criteria in Solid Tumors
RPLS: Reversible posterior leukoencephalopathy syndrome
TKIs: Tyrosine kinase inhibitors
